# An Analysis of Changes in the Physicochemical and Mechanical Properties during the Storage of Smoked and Mould Salamis Made in Poland

**DOI:** 10.3390/molecules28135122

**Published:** 2023-06-29

**Authors:** Jerzy Stangierski, Ryszard Rezler, Krzysztof Kawecki

**Affiliations:** 1 Department of Food Quality and Safety Management, Faculty of Food Science and Nutrition, Poznań University of Life Sciences, ul. Wojska Polskiego 31/33, 60-624 Poznań, Poland; 2 Department of Physics and Biophysics, Faculty of Food Science and Nutrition, Poznań University of Life Sciences, ul. Wojska Polskiego 38/42, 60-624 Poznań, Poland; ryszard.rezler@up.poznan.pl; 3 Independent Researcher, 37-500 Jarosław, Poland; krzykaw@tlen.pl

**Keywords:** smoked salami, mould salami, storage, physicochemical properties, rheological properties, texture profile analysis, sensory quality

## Abstract

The aim of the study was to analyse changes in the physicochemical, rheological, and textural properties occurring during the storage of industrially produced smoked salami and mould salami. Tests on these cold cuts were conducted on the 2nd, 15th, 30th, and 45th days of storage. There was a significant increase in the pH value of the mould salami from 5.16 on the 2nd day to 5.42 on the 45th day (*p* < 0.05). There was a downward trend in the A_w_ of the smoked salami sample from 0.892 on the 2nd day to 0.873 on the 45th day. The A_w_ in the mould salami sample decreased from 0.889 on the 2nd day to 0.847 on the 15th day and then increased to 0.871 on the 45th day (*p* < 0.05). In the first two test periods, the smoked salami was characterised by a higher modulus of elasticity value than the non-smoked salami but lower loss tangent and dynamic viscosity values. The hardness of the whole bars, as well as the hardness of the salami pieces, was affected by their storage time and the related water content. The texture test results showed that the smoked salami was more resistant to compressive force than the mould salami, which affected the sensory evaluation and ease of slicing of this type of salami.

## 1. Introduction

Salami sausages are becoming more and more popular in Poland. Salamis are typical European dry-fermented sausages, mainly made of pork meat and fat, with the addition of salt, curing agents (nitrates and/or nitrites), spices, and sugars. Starter cultures are increasingly often used as an important tool to ensure the safety and sensory quality of these products [1,2]. The production of salami is a very complex process. After filling natural or artificial casings with meat batter, the salami is subjected to fermentation and consecutive stages of drying and maturing. The drying/maturing phase is one of the most important and time-consuming stages in the production of salami because it significantly affects the colour, taste, aroma, and consistency of the end product. The aforementioned textural quality traits are determined by external factors such as temperature, humidity, and air movement speed, as well as maturation time, pH reduction, and water activity (A_w_) [3].

The safety of fermented dry sausages, as well as their resistance to changes caused by microorganisms causing their spoilage, are important issues. The safety of these meats is generally ensured by the interaction of various factors, i.e., water activity (A_w_), acidity (pH), redox potential (Eh), preservatives, and competing microorganisms such as lactic acid bacteria. Of these factors, low A_w_ and pH values are considered the most important for the inhibition and inactivation of pathogenic bacteria. At the same time, the lowering of pH and the loss of water during maturation also have fundamental influence on the textural properties of cold meats [4,5].

Salami is made from meat batter with a high content of mostly animal fat, e.g., pork salo and tallow. Commercial salami is up to 30% salo [6]. Such high fat content determines not only the palatability of salami but also its rheological and textural characteristics. From a physical point of view, meat batter is a complex multi-phase system consisting of fat, water, proteins, and other ingredients. Its properties can be significantly influenced by both technological parameters (free water, the fat–protein ratio, the water–protein ratio, the degree of comminution) and physicochemical properties. Muscle proteins are necessary to form the continuous phase of comminuted meat batters, stabilise the water and fat, and other components in the system. Salt-soluble proteins, such as myosin and its complex with actin, i.e., actomyosin, are the main group of muscle proteins used in the production of cold cuts. These proteins are characterised by well-balanced hydrophilicity–hydrophobicity and a long-chain structure. Myosin is capable of forming highly flexible gel matrices and a cohesive, rigid membrane surrounding fat globules in comminuted and emulsified meat. Protein gels have viscoelastic properties because they exhibit both liquid (viscose) and solid (elastic) behaviour. These properties are influenced by salt concentration (ionic strength), pH, and temperature [7].

The water loss which takes place during the drying/maturation process must occur at the right rate and be as uniform as possible in order to avoid case hardening, which is negative for both the safety and texture of salami. Texture parameters are essential elements determining the acceptance of food by consumers. Many authors have evaluated meat products, such as dry-fermented sausages, with the mechanical strength parameters obtained in the texture profile analysis (TPA) test as indicators of the quality of the finished product or for the selection of the best functional ingredients [8,9,10,11].

Contrary to strength analyses of the texture, dynamic mechanical analysis (DMA) enables the determination of the rheological properties and the effect of various factors (e.g., raw materials, technological factors) on the formation of their functional traits at the molecular level without destroying the measurement samples. By analysing the variability of the values of rheomechanical parameters determined during measurements, it is possible to draw conclusions about the molecular state of the system, including the stability of the protein system and emulsion [6,12]. Rheological properties are significantly related to the texture of food products, which is defined as a set of characteristics that humans can perceive with their tactile, mechanical, and, if possible, visual and auditory receptors [13].

Smoked salami and mould salami, which are produced by the largest meat processing plant in Poland, are made from the same type of meat and other technological additives. The difference between the two products consists of the fact that noble moulds are used in the production of mould salami, whereas smoking is used in the production of smoked salami. The taste and smell of both salamis are characteristic of raw, dried, and fermented sausages. Mould on the surface of the salami bar additionally gives it a delicate taste and a characteristic smell. The authors of the referenced publications did not provide any information on the influence of mould on the physicochemical properties and mechanical parameters of salami meats during storage under the conditions specified by the manufacturer. Therefore, the aim of this study was to determine and compare changes in the selected physicochemical properties occurring during the storage of two types of industrially produced salami sausages, especially their rheological properties and texture.

## 2. Results and Discussion

### 2.1. Basic Composition

The test was conducted on two types of salami made from the same raw materials and at the same essential stages of their production and maturation. Noble moulds covering the surface of the salami bar were used in the production of mould salami (MS), whereas smoking was used in the production of smoked salami (SS). As the manufacturer keeps the details of the composition of the products secret, they are not revealed in the study.

In the first period of the study, there were no statistically significant differences in the average basic chemical composition of both salamis: water 36.7 ± 0.4%, protein 25.1 ± 0.4%, fat 32.7 ± 0.4 (*p* < 0.05) (Table 1). On the 45th day of storage, there were slight but statistically significant differences. The water content in the MS sample decreased by 1.5%, whereas the protein content in the SS sample increased by 1.1%. The protein level in the MS increased by 0.9%, but the increase was not statistically significant. As the smoking process dried the surface of the salami, it may have resulted in better retention of moisture inside the finished product during storage, which was manifested by the higher A_w_ values of this salami. By contrast, this stage was absent from the MS production process. At the same time, the perforated film used for the packaging of the MS could have caused a greater loss of moisture during storage.

The content of all basic ingredients under analysis met the recipe criteria set by the salami producer, who declared a protein content of not less than 21%, a fat content of not more than 42%, and a water content of not more than 43%. There is a wide range of different types of salami, especially in Italy. They differ in the type of meat (donkey meat, pork, beef, wild boar meat, horsemeat) [14,15], the amount and type of animal fat, the degree of meat comminution, the type of spices and additives, and maturation. All these differences affect the composition, nutritional value, palatability, and, in general, the final quality of end products. For example, the basic composition of commercial Italian salami may differ significantly in the minimum and maximum content of water (24.3–53.0%), protein (24.4–37.4%), fat (12.0–29.6%), and NaCl (3.51–5.20%) [16].

### 2.2. pH Analysis

The measurements of pH in the SS sample on the 2nd, 15th, and 30th days of storage showed that its value gradually decreased from 5.88 to 5.79. However, on the last day of storage, it increased to 5.85 (Table 1). The variation in pH at different storage periods may have been caused by the proteolytic activity of staphylococcal strains in the salami [17]. It is important to note that coagulase-negative staphylococci are important microorganisms in meat products. They have some technological advantages, such as the activity of nitrite and nitrate reductase. Apart from that, they reduce the oxygen level and catalase activity, improve the colour stability, and slow down the rancidity process. A decrease in the count of staphylococci may be caused by their weaker competitiveness against lactic acid bacteria. The slower growth rate of staphylococci in salami may result from the higher acidity of the batter as well as the formation of various organic acids [3,18].

The pH values recorded in the MS on all measurement days were also below 6.0 and were consistent with the observations made by the authors of other studies [19,20]. During storage, the initial pH value increased significantly from 5.16 on the 2nd day to 5.42 on the 45th day (*p* < 0.05). The upward trend in pH observed in the MS may have been caused by the intense oxidative activity of deaminase, particularly induced by moulds [19]. The change in the pH value of the products observed at the initial period of their storage may also have been caused by the proliferation of yeasts included in the mould culture used. As yeasts proliferate, they consume lactic acid and reduce the other complex sugars in the product. Like yeasts, moulds deacidify the product.

The average pH values on all measurement days amounted to 5.84 ± 0.03 in the smoked salami and 5.29 ± 0.04 in the mould salami. They were consistent with the observations made by other authors who assessed the quality of typical Italian salami [16,19,21]. These data confirm the fact that Italian salami sausages are low-acid products, and their durability is primarily determined by reduced water activity. However, among Mediterranean products, Italian salamis are characterised by higher pH values than some traditional Spanish, Portuguese, and Greek dry-fermented sausages. The pH value of Spanish chorizo and salchichon may range from 4.32 to 5.12 [22,23].

### 2.3. Water Activity

During the storage of both salami sausages, the values of their water activity changed (Table 1). The water activity in the SS sample decreased from 0.892 on the 2nd day to 0.873 on the 45th day. The water activity in the MS sample decreased to 0.847 on the 15th day but then increased to 0.871 on the 45th day. It is noteworthy that the final A_w_ value was lower than the initial value (*p* < 0.05). The decrease in water activity in the salamis on the 2nd and 15th days can be attributed to the ongoing salami maturation process, which causes a loss of moisture [24]. However, in the two subsequent storage periods, the water activity in both samples tended to increase gradually. This may have been caused by the release of small amounts of water from the system under analysis.

The A_w_ values measured in both salami samples were in line with the values commonly observed in a normal salami maturation process lasting 45 days, where the final A_w_ value ranges from 0.81 to 0.83 [21] or may even reach 0.875 [3].

### 2.4. Rheological Properties

The production of salami is based on the batter made by mixing comminuted cured meat and fat. Curing, which takes place at the initial stage of production, significantly affects the quality, safety, and physicochemical properties of raw maturing meat products. When sodium chloride is added, it affects chemical and biochemical reactions, such as proteolysis, lipolysis, and lipid oxidation, which result in the typical texture and taste of the end products. This effect is caused by the release of taste and smell precursors from the macromolecular components of meat. The salty taste is the direct effect of adding NaCl [25], which also affects some technological characteristics, e.g., it increases water absorption and improves the gelling and emulsifying properties of meat proteins [26]. This particularly applies to the properties of muscle proteins: the interaction in the water–protein system (water-binding capacity) and the association of proteins with fat (fat-holding capacity), as well as protein aggregation (gelling capacity). These properties are interrelated and depend on various factors. As a result, the batter forms a dispersion system composed of two phases, i.e., a hydrocolloid continuous phase (it includes an aqueous, colloidal solution of muscle proteins and connective tissue and emulsions made of fat and water- or saline-soluble proteins) and a dispersed phase composed of insoluble elements of the muscle tissue and adipose tissue [27].

**Figure 1:**
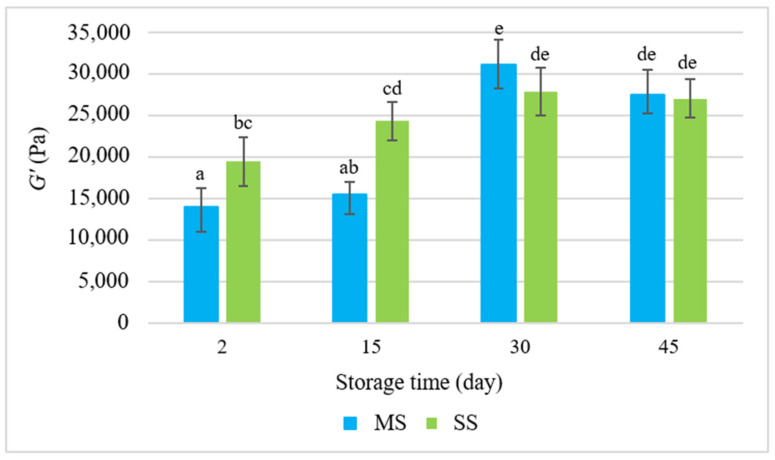
The elasticity modulus (*G*′) during the storage of the salami sausages. a–e = the mean values with the same superscript are not significantly different (*p* < 0.05; mean ± SD; *n* = 6; MS—mould salami, SS—smoked salami).

**Figure 2:**
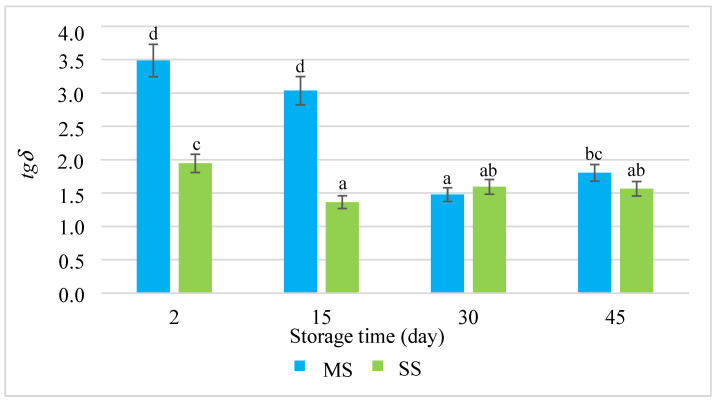
The loss tangent (*tgδ*) during the storage of the salami sausages. a–d = the mean values with the same superscript are not significantly different (*p* < 0.05; mean ± SD; *n =* 6; MS—mould salami, SS—smoked salami).

**Figure 3:**
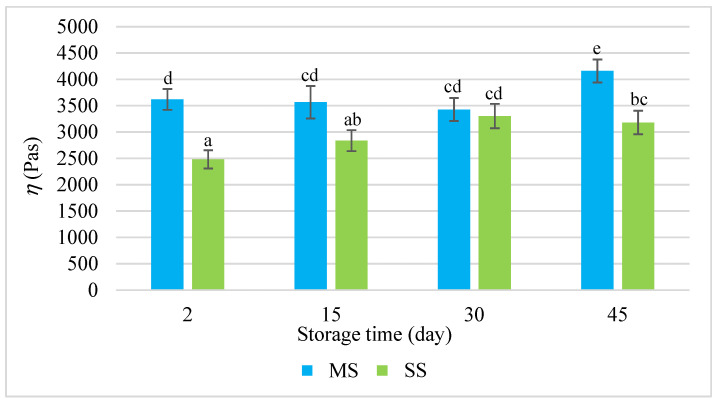
The dynamic viscosity (*η*) during the storage of the salami sausages. a–e = the mean values with the same superscript are not significantly different (*p* < 0.05; mean ± SD; *n =* 6; MS—mould salami, SS—smoked salami).

The rheological properties of dispersion systems are determined by the rheological characteristics of the continuous phase and the deformability of the dispersed phase, as well as the interactions between these phases. These changes were reflected by the results of the measurements of the rheological parameters of the salami sausages during their refrigerated storage for 45 days (Figure 1, Figure 2 and Figure 3). The analysis of the rheological determinants, i.e., the modulus of elasticity (*G*′), loss tangent (*tgδ*), and dynamic viscosity (*η*) (Figure 1, Figure 2 and Figure 3), showed that the smoking process significantly influenced these values. During the first two periods under analysis, the modulus of elasticity (*G*′) in the smoked salami (SS) was much higher than in the mould salami (MS), whereas the values of *tgδ* and *η* were lower. This means that the SS exhibited more elastic properties than the MS. The mould salami had a more compact spatial structure of the dispersion of all the ingredients of the batter. This was manifested by a higher *η* value (Figure 3) and was reflected by the adhesiveness and cohesiveness indicators. However, the high values of *tgδ* (*tgδ* > 1) led to the conclusion that the rheomechanical properties of both types of salami sausages were characteristic of viscoplastic bodies. The two types of salami differed in the values of these rheological parameters due to differences in the degree of cross-linking of their spatial protein matrices occurring as early as the production process. This resulted from the fact that the hydrocolloidal continuous phase was saturated with sarcoplasmic, myofibrillar, and connective tissue proteins to different degrees [28,29,30]. In consequence, there were different *G*′ values (Figure 1).

When salt is added during the comminution and mixing of meat, it helps to solubilise and extract myofibrillar proteins by making a sticky protein film around the formed muscle tissue particles [26]. Next, meat is acidified by the starter cultures added to it. In consequence, the dissolved proteins become denatured, and they coagulate to form a strong gel which firmly binds fat and meat. This is an additional pH-reducing factor [18]. As a result of gelation, water is released. It binds to previously inaccessible hydrophilic groups of polypeptide chains and may favour the formation of cross-links between them, thus increasing the cross-linking of the system. Undoubtedly, immediately after the production of the SS sausages, they were characterised by greater cross-linking due to the higher content of bound water in them (lower losses). This fact was confirmed by the water activity measurements on the second day of storage: SS—0.892 and MS—0.889. This disproportion in the A_w_ values in both types of sausages may have resulted from the additional technological treatment, i.e., smoking, which dried the sausage surface and effectively hindered the evacuation of water outside the system [31].

The rheological properties of emulsified systems are determined by the spatial reaction of the protein matrix to dynamic mechanical interactions. The intensity of this reaction in raw maturing cold meats is conditioned by changes in their molecular structure occurring as a consequence of biochemical, microbiological, and physical transformations. These changes take place as early as the production stage but become intensified during storage. In our study, during the storage of the salami sausages, their elastic properties increased. This fact was manifested by the increasing values of the modulus of elasticity (*G’*) in the subsequent periods of analysis, which exceeded the values measured on the second day of storage (Figure 1). The increase in the elastic properties resulted from an increase in the cross-linking of the protein matrices holding the batter dispersion components. As mentioned above, this effect is caused by biochemical (proteolytic) changes.

The approximate relationship between the modulus of elasticity of highly elastic spatial networks of polymers and the concentration of segments in these networks is determined by the relation [30]:

*G*′≅ n_s_RT (1)

where:

n_s_—concentration of segments,
R—gas constant,
T—temperature.

This relationship implies that, at a given protein concentration, the increase in elasticity of the system (hydrocolloid) at a given temperature occurs as a result of the expansion of the spatial network nodes, consisting of the binding of new molecule segments resulting from the interaction between protein polypeptide chains.

Proteolysis leads to the degradation of short-chain peptides and the release of free amino acids [32], which aggregate and form a gel with the existing ordered protein network. Intensified proteolytic effects can be observed in systems where the pH value is below the isoelectric point. In our study, starting from the 30th day of storage, the value of the modulus of elasticity *G’* in both sausages became stable. According to Spaziani et al. [18], it may have resulted from the fact that, after the first weeks of maturation of cold meats at low temperatures, fermentation is inhibited. In consequence, there is a smaller amount of extracted protein, and, thus, the expansion of the spatial network of the protein matrix is limited. During the initial storage period (up to 15 days), the value of the modulus of elasticity *G’* in the MS sausage was lower than in the SS (Figure 1). This was manifested by comparable values of both *G’* and *tgδ* (Figure 1 and Figure 2).

Throughout the storage period, the water activity A_w_ in the mould salami (MS) was lower than in the smoked salami (SS). The A_w_ in the SS was higher because the external surface of the sausage had been desiccated, which hindered the evacuation of water outside the system. The surface of the MS was inoculated with mould. As it developed on the surface of the sausage, the evaporation and loss of water from the sausage interior slowed down. As a result, the A_w_ value increased until it stabilised at 0.869 in the third period of the study. On the last day of storage (day 45), the A_w_ in both types of salami remained at a higher, but comparable, average level of 0.872. The higher content of free water in the smoked salami (SS) may have favoured the cross-linking of the extracted protein as a result of proteolytic processes and thus increased the degree of cross-linking of the spatial protein matrix. As a result, in the last period of the study, the value of the modulus of elasticity *G*′ in the SS was similar to the *G*′ value in the MS, whereas the *tgδ* values in both sausages were equal.

### 2.5. Texture Analysis

Marketing research has shown that, when consumers make purchase decisions, the texture of meat products is less important than their appearance and taste. However, this distinguishing feature is of great technological significance because it determines the possibility of slicing the product during packaging or in retail [33]. Low pH and A_w_ values are considered to be the most important for the inhibition and inactivation of pathogenic bacteria. Moreover, the decrease in pH and the loss of water occurring during the maturation of cold meats also have a fundamental influence on their textural properties [4,5].

**Figure 4:**
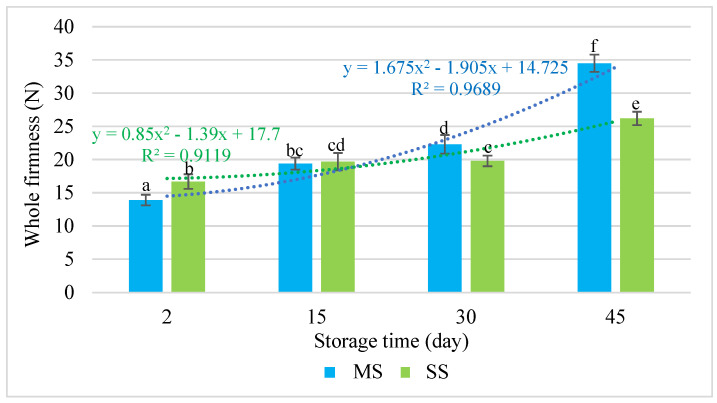
Changes in the textural properties of the whole salami bar vs. the storage time. a–f = the mean values with the same superscript are not significantly different (*p* < 0.05; mean ± SD; *n =* 12; MS—mould salami, SS—smoked salami).

The hardness of the whole salami bars was undoubtedly influenced by the storage time and the resulting loss of moisture. As the fat content in both types of salami sausages was similar, it was considered a significant factor. The content of fat, or its substitute, for example, inulin, may affect the texture parameters of a product [34]. The fitting of the experimental data to the equation with a high correlation coefficient of r = 0.984 for the MS and r = 0.955 for the SS (*p* < 0.05) showed that, for up to 30 days of storage, both types of salami were characterised by similar mechanical parameters, i.e., similar firmness in the same study period (Figure 4). In the last period (day 45), there were statistically significant differences between the whole firmness values measured in the salami sausages. In the last period under analysis, the hardness of the smoked salami (SS) increased by over 36%, whereas the hardness of the mould salami (MS) increased by nearly 60% in comparison with the hardness measured at the beginning of the study (the second day).

Our test results were in line with the findings of the study by Alamprese et al. [35], who observed an increase in the whole firmness of the pork salami during its maturation. The whole firmness of salami is closely related to its ripening time, whereas the hardness of salami pieces is higher in salami with lower moisture content but higher when meat weight loss occurs in the processing [36].

The analysis of the texture parameters of the samples cut from the inside of the salami bars showed that the SS samples were generally more resistant to compressive force than the MS samples (Table 2). They were harder on all days of the analysis. Some texture parameters of the smoked salami, i.e., hardness and chewiness, tended to decrease during the storage period. The opposite trend was observed in the mould salami. This effect may have been caused by the migration of water inside the bars during storage.

The average values of the texture parameters of the smoked and mould salamis measured in the four periods of storage were respectively as follows: hardness 128.7 N and 109.2 N, chewiness 37.3 N × mm and 34.8 N × mm. The average values of the other texture parameters remained at a similar level throughout the study period, i.e., adhesiveness 0.94 N × s (SS) and 1.13 N × s (MS), springiness 0.55 mm (SS) and 0.59 mm (MS), and cohesiveness 0.53 (SS) and 0.59 (MS). All these results of the measurements of the texture parameters indicate that the smoked salami was more resistant to compressive force than the mould salami. This seems to confirm the influence of the water content in the meat sample on its texture parameters. The analysis of the course of changes in hardness in both salami samples showed that the value of this parameter increased significantly (*p* < 0.05) in the second period of storage (the 15th day). At the same time, a decrease in the A_w_ value was observed in the same samples. The results of the measurements of the texture parameters were consistent with the direction of changes in the rheological properties.

Changes in the texture parameters occurring during the salami maturation were also observed in the sensory analysis conducted by other researchers [37]. Bañón et al. [36] also found that early hardening was detected by a panel of experts after only 30 days of storage at 10 °C, whereas the loss of juiciness was observed after 60 days following the texture instrumental data. Alamprese et al. [35] analysed salamis with different water content and found that the hardness of the products increased as the content of this ingredient decreased. When the water content was low, the elasticity values remained practically constant.

**Figure 5:**
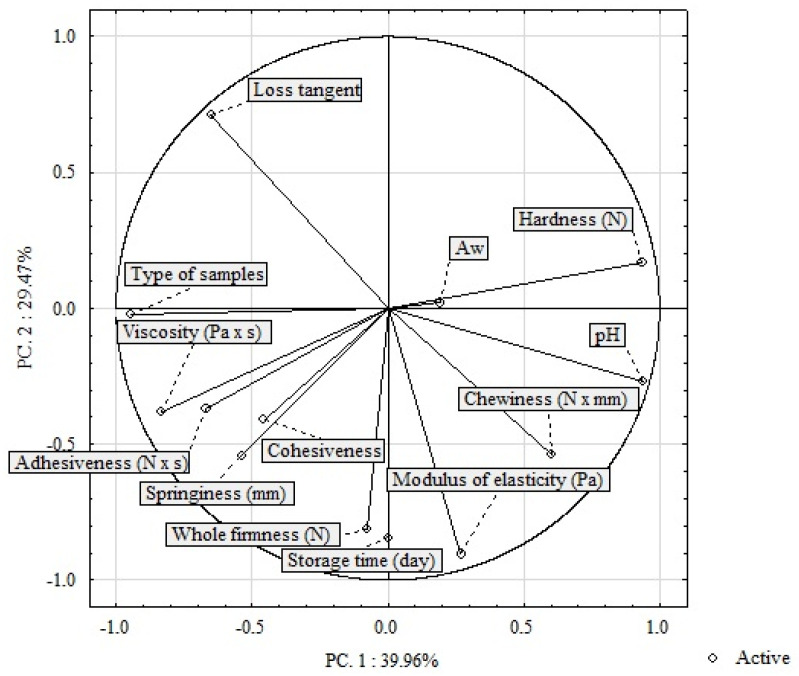
Changes in the value of selected physicochemical parameters, texture, and rheological characteristics vs. the type of salami and storage time (*n* = 448).

Two principal components were identified from the correlation matrix. They explained 69.43% of the total variability (PC1—39.96% of variability, PC2—29.47% of variability). The correlations with the input variables are shown in Figure 5. The graph shows the results of the correlation-based principal component analysis (PCA) for two types of salami samples (SS and MS) stored for 45 days. The PCA was based on all results: pH, water activity, whole firmness (N), hardness (N), adhesiveness (N × s), springiness (mm), cohesiveness, chewiness (N × mm), modulus of elasticity (Pa), loss tangent, and viscosity (Pa × s). The values of viscosity (Pa × s), adhesiveness (N × s), springiness (mm), and cohesiveness formed separate clusters, thus indicating a positive correlation with the type of sample but a negative correlation with the values of the water activity and hardness (N). The values of the loss tangent were negatively correlated with the storage time, modulus of elasticity (Pa), chewiness (N × nm), and pH.

### 2.6. Sensory Evaluation

**Figure 6:**
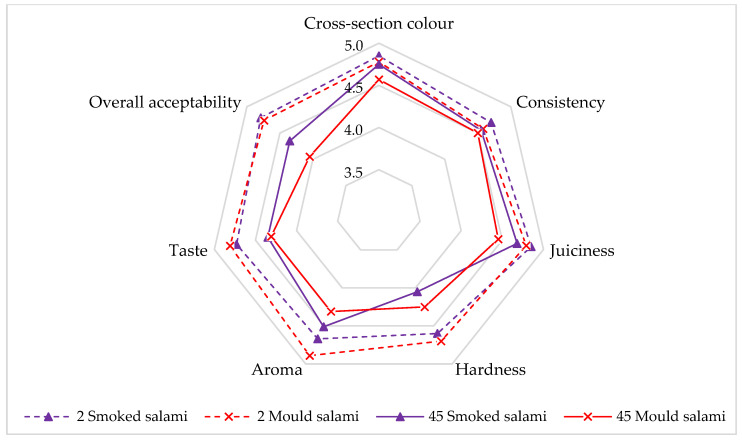
The scores of the trained panel for the intensity of the sensory characteristics of the salamis on the 2nd and 45th days of storage (*n* = 10).

As shown in Figure 6, most of the sensory characteristics were very similar to each other, especially on the second day of storage. The difference reached 0.1–0.2 points. None of the differences observed in the sensory evaluation was statistically significant (*p* > 0.05). Smoked salami was rated the highest in terms of cross-section colour (4.9) and juiciness (4.9). In the case of mould salami, the aroma was shown to be the highest-rated characteristic (4.9). Mould on the surface of the salami bar gives it a delicate taste and a characteristic smell. On the 45th day of storage, the salamis were rated slightly lower for their all sensory characteristics than on the second day of storage. The biggest, though statistically insignificant, changes were noticed in terms of hardness, taste, and overall perception regarding the smoked salami (*p* > 0.05). The difference reached 0.4 points. In addition, the instrumental analysis of the texture revealed greater hardness of the smoked salami. Higher values of these parameters affect the sensory evaluation of the texture. In the case of the mould salami, the largest decrease was found for aroma and taste at a level of 0.5 points. It was probably related to the excessive metabolic activity of the fungi during storage (excessive mould odour and odd odour) [38]. For all other sensory characteristics between samples, the differences were on the level of 0.2–0.3 points. Additionally, the overall acceptability of mould salami after 45 days of storage was rated the lowest. This was due to the deterioration of the colour of the salami surface from white to grey.

## 3. Materials and Methods

### 3.1. Description of Salami

The salami sausages were industrially produced following typical procedures with all technological parameters used in the production of these cold meats. The study was conducted on two types of salami: smoked salami (SS) and mould salami (MS). A mixture of bacterial cultures, B-LC-007 (Chr. Hansen Poland), for rapid acidification of fermented sausages, was used in the production of the salamis.

Both types of salami were mostly made from pork shoulder (85%) and salo (15%). Meat batter was comminuted through a 5 mm mesh together with spices and other additives while maintaining the batter temperature at about −2 °C. The batter was stuffed into a fibrous casing with a diameter of 70 mm. The deposition of the product at 10–12 °C lasted 2 h. Then, the surface of the bar was inoculated with a mould suspension (the mould salami only). The fermentation process at air humidity of about 92% lasted 60 h. The smoking process (the smoked salami only) at 22 °C lasted 2 h. Next, the salamis were dried at a temperature of 15 °C and air humidity of about 70% until a weight loss of 35–38%. The conditions of the maturation process were typical of ageing cured meats and lasted 21 days for the smoked salami and 35 days for the mould salami. The smoked salami was packed in plastic bags in a modified gas atmosphere. The mould salami was packed in perforated plastic bags.

The salami bars were originally packaged by the manufacturer and cold-stored at about 10 °C for 45 days. The physicochemical tests were conducted on the 2nd, 15th, 30th, and 45th days of storage (counted from the date of delivery of the material for the tests). Each time, a new, originally packed salami bar was used in the tests. Each time, two salami bars were randomly selected for instrumental analyses.

### 3.2. Chemical Analysis

#### 3.2.1. Basic Chemical Composition

The basic chemical composition of the salamis, i.e., the content of water, protein (6.25 was the conversion factor value), and fat, was measured in accordance with the methods described in the Polish Standards [39,40,41]. The basic composition was analysed on the 2nd and 45th days of cold storage.

#### 3.2.2. pH Measurement

The salami samples (10 ± 0.01 g) were diluted with deionised water (1:10) and homogenised. The pH value was measured with a portable digital HI 99161 m (Hanna Instruments, Eibar, Spain) equipped with an FC2023 glass electrode. The electrode was calibrated with pH 7.0 and 4.0 buffers (Merc, Frankfurt, Germany).

#### 3.2.3. Water Activity Measurement

The water activity (A_w_) was measured with a HygroPalm 23-AW (Rotronic Instruments Ltd., Crawley, UK). Samples used for water activity measurement at 20 °C were minced into smaller pieces (approx. 3–5 mm). The sample dishes were filled as evenly as possible, at least two-thirds full, and, subsequently, the lid was closed. Before each measurement, the chamber was dried to a water content of 0.1.

#### 3.2.4. Rheological Properties

Dynamic mechanical analysis (DMA) was used to study the rheological properties of the salamis. This is a non-destructive analytical method, meaning it can be used to determine the viscoelastic properties of foods without altering the material structure. This method is based on the fact that, for viscoelastic materials, the resultant material sinusoidal stress response lags in phase/time from an applied sinusoidal strain by an angle *δ*. In such experiment, a sinusoidal oscillating stress with a frequency *f* is applied to the material, time differences between the oscillating stresses are measured. The generated stress is expressed in terms of the elasticity modulus *G*′ (Pa), loss modulus *G*″ (Pa), loss tangent *tgδ*, and dynamic viscosity *η*. The macroparameters (*G*′, *tgδ*, and *η*) describing the elasticity of the tested systems were the result of measurements made with a dynamic mechanical rheological analyser DMWT (COBRABiD-Poznań, Poland). *G*′ is related to the part of the potential strain energy which is retained during periodic deformations. The loss tangent (*tgδ*) is a measure of internal friction and determines the relative amount of energy dissipated in the material during one deformation cycle [12]. The tests were made in a plate–plate measuring system (*ϕ* = 0.03 m, *d* = 0.002 m) at room temperature (20 °C). A salami specimen in the form of a circular disk was sandwiched between parallel plates. The bottom plate was fixed. To avoid the sliding effect, the surfaces of both plates (bottom and top) were serrated. The top plate oscillated behind a frequency of 2.6 Hz. The temperature of the chamber and the measuring plate was measured with an accuracy of ±0.2 °C. The analysis of rheomechanical parameters was conducted in the linear range of the viscoelastic properties of each sample.

#### 3.2.5. Texture Analysis

The texture of whole salami bars and cut salami samples was analysed with a TA-XT2i texture analyser (Stable Micro Systems, LTD., Surrey, UK). A 25 kg load cell was applied to obtain force–time deformation curves. The data acquisition rate was 200 PPS, and the applied force was 0.20 N. The texture of the salamis was analysed at about 20 ± 1 °C.

#### 3.2.6. Compression of Whole Salami

The hardness of the salami bars was measured with a spherical probe (diameter 5 mm) by penetrating the bars to a depth of 20 mm. The head of the texture meter moved at a speed of 1 mm/s. Each time, the bars were penetrated at eight different points along the main axis. As a result, whole firmness (N) was determined.

#### 3.2.7. Compression of Salami Pieces (TPA)

Cylinder-shaped samples with a diameter and height of 20 mm were cut out of the sausage bars for texture profile analysis (TPA). The salami samples were compressed twice to 50% of their original height. The relaxation time between the compression cycles was 0.1 s. The head moved at a speed of 5 mm/s.

#### 3.2.8. Sensory Analysis

The end products were evaluated by a 10-member trained panel (seven women aged 30–40 and three men aged 36–40) according to the requirements of the standard [42]. The intensity of the selected characteristics of the products was assessed quantitatively according to a predetermined scale based on the scaling method described in the PN-ISO 4121:1998 standard [43]. The sensory panel evaluated two coded samples (the smoked salami and mould salami) one by one in a random order. The following sensory characteristics of the end products were evaluated: cross-sectional colour, consistency, juiciness, hardness, aroma, taste, and overall acceptability. Individual sensory characteristics were evaluated according to a five-point scale with the following degrees of intensity: very attractive, distinctive, standard (5 points), attractive, with small deviations (4 points), average, with noticeable deviations (3 points), unsuitable, with considerable deviations (2 points), and unacceptable (1 point). Sensory evaluation was conducted on the 2nd and 45th days of storage of the salami.

#### 3.2.9. Statistical Analysis

The results of the measurements of the basic chemical composition, pH, water activity, rheology and texture were analysed statistically with SPSS software v. 13.1 (StatSoft, Tulsa, OK, USA) and expressed as mean ± SD. Differences were considered significant at *p* < 0.05. The group mean was compared with Tukey’s post hoc test. Multivariate analysis of variance (MANOVA) was used to assess the influence of the type of salamis and storage on the physicochemical properties, water activity, and pH. Principal component analysis (PCA) was applied as the first step of data analysis to visualise information and detect patterns in the data.

## 4. Conclusions

The study characterised two types of salami made in Poland. The rheomechanical properties of the sausages used in the tests were conditioned by biochemical and microbiological transformations, as well as by the effect of changes in their molecular structure which occurred both during the production maturation and during storage. These changes affected such characteristics as the water content, pH, water activity, and mechanical parameters. The changes observed in the salami samples during their storage were mostly caused by the smoking process applied in the production of one salami variant (SS). The smoking process caused some weight loss induced by the drying of the surface of the salami bar. As a result, there was a lesser loss of water and lower dynamics of changes in the A_w_ value in the smoked salami than in the mould salami. It is also likely that the amount of water loss from the samples of mould salami was influenced by the perforated package. In the last period of storage, the mould salami had lower water content, lower pH, and lower A_w_. This may result in greater durability and health safety of this type of salami. The aforementioned physicochemical parameters influence the mechanical properties of salamis. Both types of salamis exhibited the rheomechanical properties characteristic of elastoplastic bodies. The initial values of the rheological determinants of the salamis were different due to the different technology applied (smoking). However, after a longer period of storage, they became equal. The instrumental analysis of the texture revealed greater hardness and chewiness of the smoked salami. These results correlated with the sensory analysis. The higher values of these parameters affect the sensory evaluation of the texture and the ease of slicing of this type of salami.

## Figures and Tables

**Table 1:** The basic chemical composition (%) and results of the physicochemical analysis of the salami during storage.

Type of Sample and	Moisture (%)	Protein (%)	Fat (%)	pH	A_w_
Storage Time (Day)					
Smoked salami (SS)					
2	36.9 ^a^ ± 0.5	24.7 ^b^ ± 0.3	32.8 ^a^ ± 0.4	5.88 ^a^ ± 0.04	0.892 ^a^ ± 0.004
15	-	-	-	5.82 ^ab^ ± 0.03	0.869 ^b^ ± 0.005
30	-	-	-	5.79 ^b^ ± 0.03	0.872 ^b^ ± 0.006
45	36.1 ^a^ ± 0.4	25.8 ^a^ ± 0.4	33.1 ^a^ ± 0.6	5.85 ^ab^ ± 0.03	0.873 ^b^ ± 0.004
Mould salami (MS)					
2	36.3 ^a^ ± 0.3 ^a^	25.4 ^ab^ ± 0.5	32.6 ^a^ ± 0.5	5.16 ^d^ ± 0.04	0.889 ^a^ ± 0.003
15	-	-	-	5.17 ^d^ ± 0.03	0.847 ^c^ ± 0.004
30	-	-	-	5.48 ^c^ ± 0.05	0.869 ^b^ ± 0.003
45	34.8 ^b^ ± 0.4	26.3 ^a^ ± 0.6	33.5 ^a^ ± 0.6	5.42 ^c^ ± 0.04	0.871 ^b^ ± 0.004

^a–d^ = means in the same column with different superscript letters indicate a significant difference at *p* < 0.05 (mean ± SD; *n =* 6).

**Table 2:** Texture profile analysis of the salami depending on the storage time.

Texture Parameters	Type of Salami	Storage Time (Day)			
		2	15	30	45
Hardness (N)	SS	138.0 ^d^ ± 4.9	131.8 ^c,d^ ± 6.1	116.8 ^b^ ± 5.8	128.1 ^c^ ± 6.1
	MS	99.4 ^a^ ± 3.9	118.2 ^b^ ± 4.2	110.0 ^b^ ± 5.7	109.2 ^b^ ± 2.6
Adhesiveness (N × s)	SS	0.98 ^b^ ± 0.01	0.92 ^b^ ± 0.15	0.94 ^b^ ± 0.13	0.91 ^b^ ± 0.19
	MS	1.13 ^a,b^ ± 0.09	0.96 ^b^ ± 0.10	1.31 ^a^ ± 0.18	1.13 ^a,b^ ± 0.14
Springiness (mm)	SS	0.53 ^a,b^ ± 0.02	0.56 ^b^ ± 0.02	0.57 ^b,c^ ± 0.02	0.55 ^a,b^ ± 0.03
	MS	0.61 ^d^ ± 0.03	0.52 ^a^ ± 0.03	0.61 ^c,d^ ± 0.02	0.62 ^d^ ± 0.03
Cohesiveness	SS	0.52 ^b^ ± 0.02	0.52 ^b^ ± 0.01	0.54 ^b,c^ ± 0.01	0.52 ^b^ ± 0.02
	MS	0.57 ^c^ ± 0.02	0.49 ^a^ ± 0.	0.56 ^c^ ± 0.01	0.56 ^c^ ± 0.02
Chewiness (N × mm)	SS	38.4 ^b^ ± 3.2	38.5 ^b^ ± 2.6	35.7 ^a,b^ ± 1.9	36.7 ^a,b^ ± 3.6
	MS	34.1 ^a^ ± 2.0	31,1 ^a^ ± 2.1	36.4 ^a,b^ ± 3.4	37.7 ^a,b^ ± 2.5

^a–d^ = means in the same row with different superscript letters indicate a significant difference at *p* < 0.05 (mean ± SD; *n* = 8; SS—smoked salami, MS—mould salami).

## Data Availability

All data are contained within the article.

## Sample Availability

Samples of the compounds are available from the authors.
